# Risk Perception and Ethnic Background in Construction Workers: Results of a Cross-Sectional Study in a Group of Trainees of a Vocational School in Italy

**DOI:** 10.3390/ejihpe11010008

**Published:** 2021-01-26

**Authors:** Federico Ricci, Giulia Bravo, Alberto Modenese, Fabrizio De Pasquale, Davide Ferrari, Massimo Bello, Gianluca Favero, Sergio Soddu, Fabriziomaria Gobba

**Affiliations:** 1 Department of Biomedical, Metabolic and Neural Sciences, University of Modena & Reggio Emilia, 41125 Modena, Italy; federico.ricci@unimore.it (F.R.); giulia.bravo@uniud.it (G.B.); alberto.modenese@unimore.it (A.M.); bello_massimo@yahoo.it (M.B.); 2 Department of Medicine, University of Udine, 33100 Udine, Italy; 3 Department of Public Health, National Health Service, 41126 Modena, Italy; f.depasquale@ausl.mo.it (F.D.P.); da.ferrari@ausl.mo.it (D.F.); 4 Observatory for the Prevention, 33074 Fontanafredda, Italy; gianlucafavero@gmail.com; 5 Department of Public Health, National Health Service, 40068 San Lazzaro di Savena, Italy; sergio.soddu@ausl.bo.it

**Keywords:** risk perception, ethnicity, construction sector, safety climate, perceived behavioral control, attitude towards safe actions

## Abstract

Risk perception can be influenced by cultural background. The study aims to evaluate risk perception, considering different ethnicities of construction workers from vocational schools in Italy. We administered a questionnaire investigating four different dimensions: Perceived behavioral control (PBC), Danger perception (DP), Safety climate (SC), and Attitude towards safe actions (ATSA). 562 workers answered: 72.4% from Italy, 14.2% from eastern Europe, 9.4% from Balkans, and 3.9% from North Africa. The participants indicated quite low control, attributable to the haste in performing the job. The workers perceived their specific job tasks as riskier compared to the tasks of their colleagues. They reported as fundamental the respecting of safety rules, but indicating that supervisors do not adequately promote safety behaviors. Finally, construction workers judged as “brave” the colleagues working without protective equipment. When compared to Italians, North Africa workers showed a lower perception of the possibility to control their safe behaviors (*p* = 0.040), while both eastern Europeans and Balkan obtained higher scores at the ATSA dimension, indicating a kind of fatalistic acceptance of the risky situations at work. Eastern Europeans also showed a lower perception of the dangers (*p* = 0.002), while Balkan demonstrated a perception of SC even better than the Italian group (*p* = 0.005).

## 1. Introduction

Data indicate that the construction industry accounts for about 25% up to 50% of the fatal work-related accidents in industrialized countries [1]. In Europe, during the year 2018, 3332 fatal accidents at work were reported, with an increase of the deaths compared with 2017; about 20% these fatal accidents involved the construction sector [2]. 

The construction sector in the industrialized countries is typically an occupational sector where quite a relevant number of migrant workers are employed: the International Labour Organization estimates there are about 163.8 million migrant workers, most of them employed as manual laborers in the construction sector [3]. Various reports indicate that migrant workers are more exposed to risky situations when compared to natives, and it is also reported that they have usually lower wages and longer working hours compared to natives. These factors may play a role in their more frequent involvement in work-related adverse events, such as occupational injuries and diseases [4,5,6,7]. 

### Literature Review, Hypothesis Development and Objectives of the Study

Language and culture barriers are considered additional risk factors possibly affecting the relations with other social determinants of health and occupational risks for workers with different ethnic backgrounds and migrant workers [8,9]. The perception of these risks, i.e., the subjective judgment on the probability of experiencing a negative event [10,11], has shown cross-cultural differences, possibly determined by several factors including individual risk attitude, risk sensitivity, and specific fears [11,12].

Casey et al. found that the national culture of a group of Anglo and Southern Asian workers in a multinational oil and gas company could explain the differences in their safety-related perceptions and safety compliance [13]. In particular, the authors reported that supervisor production pressure was negatively related to willingness to report errors, but did not predict safety compliance behavior, and these relations were stronger among South Asian employees [13]. Pressure to work was found to be a specific factor influencing injury and hazard reporting in a group of Hispanic immigrant dairy workers [14]. Moreover, an adequate risk perception at work, and in particular the appropriate knowledge of the possible occupational hazards and dangers present at the workplace, is important, as it is associated with a better physical and mental health of the workers [15]. Another factor implicated in the extent of exposure to occupational risks for migrant workers is the perception of control, found to be an important predictor [16].

Based on these findings, various studies have suggested to give an adequate consideration to workers’ perception of environmental and occupational health and safety (OHS) issues, weighting the role of socio-demographic characteristics, including ethnicity, when designing research studies and developing preventive interventions, as safety trainings [17,18]. 

There are few examples of these interventions in scientific literature: Vela Acosta et al. reported of an interdisciplinary participatory approach, integrating educators and researchers with a community advisory board to develop a specific bilingual OHS intervention for Hispanic farmworkers in Texas [19]. The completion of the curriculum improved the safety risk perception and safety behavior of the workers [19].

Unfortunately, to the best of our knowledge no published reports on the results of preventive training interventions that have specifically considered the ethnic background of construction workers are available. One of the possible reasons is that, most often, construction companies are small or very small, and it can be a problem to give to the workers an adequate training, taking into account their languages and ethnic backgrounds [20].

**Figure 1:**
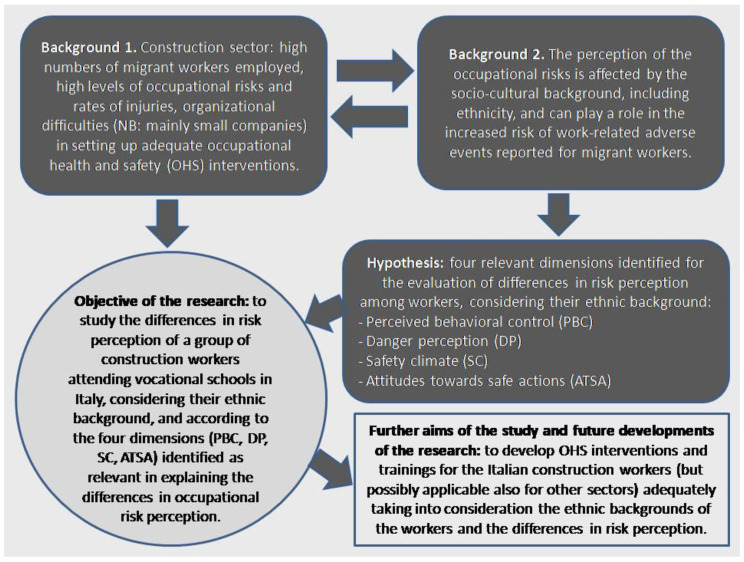
Development of the research work of the study.

Considering these premises, we started from the hypothesis that the study of the ethnic background and cultural differences is helpful for a better understanding of the modalities and of the reasons of the safety behaviors adopted by the workers at the workplace, as cultural values affect the way people think and behave when facing safety-related problems [8,9,10,11,12,13,14,15,16,17,18,19]. We, therefore, decided to evaluate the relationships between risk perception and socio-cultural factors, including ethnic background, as safety interventions that do not consider socioeconomic, cultural and demographic aspects are less likely to have a significant impact [21]. We addressed construction workers because, as previously stated, this is a sector where a lot of migrant workers are employed, with an increased risk of occupational injuries, and where it can be difficult to organize effective trainings for the workers. To fill this gap, we decided to study the differences in risk perception, for a further development of better preventive initiatives (Figure 1).

In this study, it was assumed that risk perception concerns the subjective judgment that people express about the characteristics and severity of a risk, weighting positive and negative consequences of dangers at their workplace and making decisions on how to deal with OHS issues [10,11,22]. Moreover, for the purposes of this manuscript, an ethnic group is defined as, “a named social category of people based on perceptions of shared social experience or one’s ancestors’ experiences” [23].

Accordingly, the results of the investigation of different precursors of safe behavior among construction workers with different ethnic backgrounds are presented here, based on the following constructs, identified through a factorial analysis in a previously published work [24], and recently applied also to other OHS issues in different sectors [25]:

- *Perceived behavioral control* (PBC), according to the theory of planned behavior [26]: this dimension indicates a measure of the perceived grade of difficulty attributed by a person to the decision of taking a specific action in order to obtain a specific result (i.e., problems experienced by construction workers in terms of complying with the safety rules at work).
- *Danger perception* (DP), that is a measure of the perceived level of consciousness in recognizing a job as intrinsically dangerous. This reflects the fact that construction sector can be considered as intrinsically dangerous, with several occupational risks of higher levels compared to other sectors.
- *Safety climate* (SC), representing the way by with safety rules are interiorized in the organizations, and, accordingly, perceived by the workers through their relations with colleagues and supervisors, in the same way as “subjective norms” are defined in the theory of planned behavior [26]. Safety climate can be influenced by the actions of other people inside the work team, in particular if they share the same socio-cultural background.
- *Attitude towards safe actions* (ATSA), a dimension designed according to the three components of attitudes identified in 1960 by Rosenberg and Hovland [27]: cognitive, affective, and behavioral. Accordingly, workers may act, or not act, in terms of safety behaviors, depending on their beliefs, emotions and personal leanings.

Evaluating these precursors of safe behaviors at work, the aim of the research was the studying of risk perception of a group of construction workers in relation to their ethnicity, in order to build more accurate safety trainings and preventive interventions in the construction sector, potentially applicable also to other occupational activities.

## 2. Materials and Methods

### 2.1. Study Population and Data Collection

The study population is constituted by a convenience sample of Italians and immigrants, recruited in vocational schools for the training of construction workers. The specific types of vocational schools considered in this study are called in Italy “Scuole Edili” (i.e., Building Schools). These institutes are the main reference for the vocational and safety training of the construction workers, and are affiliated to “FORMEDIL”, which is the National Joint Association for training in the building sector. 102 building schools are included in the FORMEDIL network, providing more than 11,000 courses to approximately 142,000 participants every year [28]. As OHS training is mandatory in Italy, and as Building Schools are the most widespread institutes for delivering it, we decided to recruit the study population in four different vocational schools from two areas of Italy, two institutes representative for the North of the country (Emilia Romagna region) and two of the South (Sardinia region).

The only inclusion criteria were that of being a construction worker, having attended a course at one of the above-mentioned Building Schools, having an age between 16 and 65 years old and having Italian, eastern European, Balkan, or North Africa origins (NB: ethnic groups aggregated according to ILO [3]). We excluded workers from other continents (apart North Africa) or from other parts of Europe as they usually represent in the construction sector in Italy an extremely low percentage of the non-Italian workforce (<5% of all the foreign workers employed [29]), and therefore it would be very difficult to reach a statistically significant sample of workers.

Another exclusion criteria was that of the language comprehension: as for the participation in the study it was required to fill in a written questionnaire, the workers needed to have a minimal reading ability, corresponding to a language comprehension according to the Common European Framework of Reference for Languages (CEFR) of at least a B1 level (i.e., “Can read straightforward factual texts on subjects related to their field of interest with a satisfactory level of comprehension”), and accordingly the subjects showing a level of A1 or A2 [30] were excluded.

The data for this research have been collected for all the workers participating to the trainings organized by the vocational schools that joined the research project proposed to evaluate risk perception in Italian and foreign workers of the construction sector [31]. The participation in the study of the workers was on a voluntary basis: before the beginning of the training, an expert in OHS explained to the workers the aims of the project and gave all the indications for the participation in the research.

### 2.2. The Questionnaire

Before the beginning of the training sessions, in order to avoid any influence on the answers of the responders, the workers were asked to fill-in a detailed questionnaire, that was specifically elaborated for the purposes of evaluating risk perception of construction workers, considering their ethnic backgrounds [24]. The first version of the questionnaire counted 77 items divided in three sections. Sections “A” and “B” (NB: available as Supplementary Materials) are the general parts of the questionnaire, aimed at collecting socio-demographic information of the workers and data related to the occupational history and to the OHS risks. Among the general information collected there are the country of origin and the subjective evaluation of the ability in understanding and speaking the Italian language, the age and the educational level (classified as following: none, primary, high, university). Considering occupation, data on the professional qualification (classified as following: construction manager, foreman, specialized worker, generic worker, no qualification, other qualification) were collected, as well as on the type of work contract (classified as following: permanent employee, seasonal worker, private craftsman collaborating with a construction company, unemployed, other type of contract), on the specific job tasks performed (e.g., mason, carpenter, etc.) and on some general information on the history of work-related injuries and on previous OHS trainings (e.g., ever experienced an occupational injury; ever participated in an OHS training). These two sections were not cut in the final version of the questionnaire administered for the present analysis, while from the section “C” of the survey 34 items have been cut, resulting in a short edition of only 12 items applied in this study (Table 1) [24]. The section “C” of the questionnaire is the one specifically designed for the evaluation of the risk perception of the construction workers taking into account their ethnic backgrounds. The process by which this section was constructed is described in detail in our previously published methodological work [24]. Briefly, starting from the original version of the section “C” prepared by a multi-disciplinary group of OHS experts, adopting short and simple sentences, the final version was elaborated after a pilot administration in a sample of 527 construction workers with different ethnic background and a specific analysis of their answers [24]. The analysis done were aimed at finding the unnecessary and ambiguous items, evaluating the internal consistency of the survey, to finally produce a short and coherent version, in which the items could be traced back through a factor analysis to four specific dimensions, identified as the precursors of risk perception in Italian and immigrant construction workers in the considered pilot sample: PBC, DP, SC and ATSA [24]. Considering this, for the present research a questionnaire counting three sections and 43 items in total has been administered to the participating workers. To each item of the section “C” it was asked to answer on an 11-point Likert scale (from 0 = Absolutely disagree, to 10 = Absolutely agree).

### 2.3. Data Analysis and Statistics

All the data collected with the questionnaires have been computerized by the authors (G.B., A.M., F.D.P., and M. B.), assigning a progressive alphanumeric code to each questionnaire and therefore building an anonymous electronic database, using the statistical software package SAS version 9.4 for Windows [32]. The first step of data analysis involved the description of the study population, mainly examining the answers to the sections “A” and “B” of the questionnaires and so describing the general characteristics of the participants. Common descriptive statistics techniques have been applied to analyze the answers of the recruited workers, including the evaluation of the frequencies of the different predetermined answers, creating tabs for categorical variables, and the calculation of mean values and standard deviation (SD) for the answers resulting in continuous variables. To assess whether the groups of the workers differed for their general characteristics according to their ethnicity we applied the Student *t* Test in case of continuous variables and the Chi-Square Test for the categorical ones, considering significant the differences with a *p* value < 0.05 [33]. The second step of the data analysis included the evaluation of the answers given to the section “C” of the questionnaire, i.e., the one specifically aimed at evaluating the risk perception of the construction workers. First of all, the mean scores (and the SD) for each item was calculated, and the items were grouped according to the four dimensions identified as indicators of risk perception in construction workers: PBC, DP, SC, and ATSA. Then, the results of the section “C” administration were analyzed also according to the three different ethnic groups identified [3], i.e., workers from eastern Europe, from Balkans and from North Africa, using the answers of the Italians as a reference. The associations between the scores obtained in the different four dimensions and the ethnic groups of the workers have been evaluated, through a multivariate logistic regression model [34]. The model was adjusted considering the following confounding variables: age, personal experience of an occupational injury, being an eyewitness of a work-related accident, professional qualification, and work contract.

## 3. Results

### 3.1. Description of the Study Population

The sample is composed of five hundred and sixty-two (562) workers from different ethnic groups. Italian workers are the most represented group (n = 407; 72.4%), while the 27.6% of the sample includes various ethnicities, aggregated in three major groups, according to ILO [3], as following:

- Eastern Europe (Lithuania, Poland, Moldova, and Romania), n = 80, 14.2%;
- Balkans (Albania, Croatia, Kosovo, and Serbia), n = 53, 9.4%;
- North Africa (Algeria, Egypt, Morocco, and Tunisia), n = 22, 3.9%.

In the whole sample, the age varies between 16 and 65 years, with a mean value of 36.9 (±11.8). Italians are the older group, with a mean age of 38.3 (±12.3) years compared to others (33 years old), and the difference is significant (*p* < 0.0001). Considering educational level, the majority of the subjects (49%) attended only primary school, followed by high school (46.1%). Regarding the job position in the construction sector, the 34% of the workers are “generic construction workers” (e.g., laborers and generic masons) and the 30% are “specialized workers” (e.g., carpenters, crane conductors, etc.). Finally, considering the type of work contract, the 63% of the participants are permanent employees. Among the three ethnic groups identified, we found no significant differences according to the variables “job position” (*p* = 0.7697) and “work contract” (*p* = 0.0891), while the difference was significant comparing the migrant workers group overall with the Italians, for these variables as well as for the educational level (*p* < 0.0001) (Table 2).

Considering the characteristics of the study population according to the information on work-related injuries and on their prevention, the 30.8% of the workers reported a personal experience of occupational injuries, and of these workers the 83.2% were from Italy (*p* < 0.001). On the other hand, only 19 workers, the 3.4% of the total, reported an experience as eyewitness of an occupational injury happened to a colleague, and the Italians represented only the 37% of these cases (*p* < 0.001). Regarding the information received on the prevention of the risks of occupational injuries, the 71.9% of the sample judged these notions as adequate, and also in this case Italians were less represented in this group compared to the distribution of the whole sample, being only the 54% (*p* = 0.0170) (Table 3).

### 3.2. Results of the Analysis of the Four Dimensions of Risk Perception in Construction Workers

Considering the analysis of the four dimensions of risk perception and safe behaviors identified by the section “C” of the questionnaire, the results of the answers given by the sample of construction workers are shown in Table 4. The scores of the answers are evaluated on a 10-points Likert scale based on the level of agreement with the statement proposed in the items of each dimension (0 = full disagreement, up to 10 = full agreement).

Summarizing the main findings presented in Table 4, it should be underlined that:

- According to the “Perceived Behavioral Control” dimension, the participants indicated quite high score, representative of a lower perception of control, mainly attributable to the haste in performing the job, as a consequence of supervisors’ pressure, but also to the fear of losing the job and to the tiredness cause by the work.
- Considering the dimension “Danger perception”, the responders indicated to perceive their specific job tasks as riskier compared to the tasks carried out by other colleagues and superiors, but in general they do not judge the work activities in the construction sector to be particularly dangerous, excluding the possibility of serious harms.
- For the dimension of the “Safety climate” the participants highlighted the fundamental importance of respecting the rules, as e.g., safety requirements, while it is quite low the score attributed to the question investigating the role of the supervisor in promoting an adequate safety climate in the company, with examples of safety behaviors and respecting safety rules.
- Finally, regarding the dimension “Attitude towards safe actions”, the answers of the workers indicated a quite ambivalent attitude, both demonstrating a cautiousness related to the knowledge of the extreme severity of the possible occupational injuries that may happen, but also judging as “brave” the colleagues or superiors who are able to work without adequate protective equipment.

Considering now the relations between the four dimensions of risk perception and the ethnic background of the workers, some significant associations between the ethnic group and the scores obtained in the dimension, when compared to Italian workers, have been identified: the odds ratios (OR) obtained with the adjusted multivariate logistic regression model are presented in Table 5.

In particular, when compared to Italians, North Africa workers had significantly higher scores at the PBC dimension (OR = 1.752, *p* = 0.040), perceiving more, compared to Italians, the haste of the job and the fear of losing it, as well as the pressure of the supervisor. Workers from eastern Europe showed a lower awareness about the possible danger situations and risky exposures at the workplace compared to Italians (OR = 0.635, *p* = 0.002*), and higher scores in the dimension ATSA, indicating that they felt as “brave” when working without adequate protections (OR = 1.441, *p* = 0.011). Finally, also Balkan workers obtained higher values at the ATSA dimension (OR=1.601, *p* = 0.007), but they are the only group reporting a significant better perception of the safety climate compared to the Italians (OR = 1.611, *p* = 0.005) (Table 5).

## 4. Discussion

The aim of this study was to evaluate the risk perception in construction workers and its relation with different ethnic groups, in order to address properly tailored safe behaviors trainings and interventions in the workplace.

Risk perception can determine an impact on the safe behaviors, resulting in a higher or lower probability of accidents and injuries [35]. In the construction sector, the poor ability to identify the risks is a fundamental problem in the safety management. In fact, a good perception of the risk allows to adopt adequate protective behaviors, and, at the same time, an increase in knowledge produces a better risk perception and contributes to an adequate safety climate [36].

According to the Theory of Planned Behavior [26] and to the components of attitudes [27], human action is the result of three conditions that, in the field of health protection, could be defined as attitude towards safe actions, safety climate, and the awareness of being able to implement a safe behavior (i.e., perceived behavioral control) [26,27]. Accordingly, this research investigated three factors included in these theories, that promote the intention to implement safe behaviors, and a fourth construct concerning the knowledge of the condition of danger in the working environment (danger perception).

The main findings, in the whole sample examined, indicate that working conditions such as haste, fatigue, and fear of losing the job, negatively affect the perceived control and the compliance with safety rules at work. Moreover, the participants considered their specific job tasks as riskier compared to the activities carried out by other colleagues in the construction site, but nevertheless they underestimated the possibility of serious harm. These results may prove that the construction workers of the convenience sample studied are kind of used to deal with dangerous conditions, with a limited perceived control over them. Furthermore, they reported an ambiguous attitude towards the perception of the relevant OHS aspects, including the norms and the information received for the prevention of the injuries. In fact, despite they declared as being fundamental the compliance with the safety requirements, they also reported that their supervisors did not adequately promote the respect of the safety rules at the workplace. These ways of perceiving the occupational risks may have determined in the examined workers a kind of ambivalent attitude towards safe actions: in fact, even if they reported to know that they are exposed to a high risk of injuries, they recognized as an expression of “bravery” the condition of working with an inadequate adoption of protective equipment. These results can be also seen as a confirmation of the “chain of social effect” theory, described by Meliá et al. [37]: when the supervisors do not adequately promote the safety climate at work, the workers tend to develop a favorable attitude towards the non-use of protective devices. 

Considering now the relations between the ethnic groups and the four dimensions of risk perception, the data collected point out some significant characteristics for each of the ethnic sub-groups investigated, when compared to Italian workers.

- Perceived Behavioral Control dimension showed higher scores for North African workers, indicating difficulties in complying with safety rules due to external factors that they are not able to control, as the fear of losing the job or the feeling of pressure and haste at work.
- Construction workers from eastern Europe indicated that they perceive the construction sector not as highly dangerous as it is perceived by the Italians. Probably as a consequence, they also reported to identify as “bravery” the attitude of working with inadequate adoption of protections, looking at the possibility of injuries in a kind of fatalistic way.
- Balkan workers are from regions not so much different compared to eastern Europe: probably these ethnic groups may share some aspects of their safety behaviors, as confirmed by their attitudes towards safe actions, which, as happened also for eastern Europeans, showed a habit possibly representing a condition of passive acceptance of the eventuality of a work accident, and considering the workers reporting an inadequate use of protections as courageous. On the other hand, differently from eastern Europeans and North African workers, Balkan workers were the only group scoring higher at the safety climate dimension, when compared to Italians. This means that, in general, they perceived a good respect of the safety rules at work and that they trusted their supervisors, promoting adequate safety behaviors.

Regarding the possible implications of the results obtained, a first consideration needs to be related to the fact that various working factors can negatively influence the adequate perception of the risks at the workplace, possibly increasing the risk of accidents: among the main factors, in particular the role of team leaders and supervisors emerges from this study as fundamental for an adequate promotion of safety culture at the workplace in the construction sites. Moreover, when planning preventive interventions for the health and safety at work in the construction sector, a particular attention should be given also to the ethnic background of the workers, not only for the well-known problems of the language, socioeconomic, and cultural barriers and habits [38], characteristic of some specific groups, but also considering that different ethnicities and culture may result in significant differences in the perception of the occupational risks and, more generally, of the safety culture of the company. This may impact the adoption of the safe behaviors by the workers, as well as their responses to specific modalities of delivering safety information and of promoting the safety climate.

The research performed has also some limitations. First of all, it cannot be excluded a possible selection bias, as the convenience sample investigated was recruited on a voluntary basis from workers participating to various trainings organized in a few Italian vocational schools. In any case, we believe that our sample can be considered representative of the Italian construction sector as:

- In Italy, at least a minimal sixteen hours certified OHS training is mandatory for all the construction workers prior to have access to a construction site, and this training has to be repeated at least every five years with a six hours recall session [39]. We are aware that it is possible that the specific paths of the trainings of the included workers differed significantly, as vocational schools organize several different trainings, also according to the role of the workers and to particular responsibilities they may have in terms of OHS (e.g., representative of the employees for the safety or workers assigned to the first aid procedures, respectively requiring additional 32 and 16 hours trainings) or according to the specific technical qualifications (e.g., for the installation of scaffolding, requiring additional 32 hours of training). Nevertheless, even if with possible differences in the courses followed, all the construction workers in Italy attend, at least for a short period, a specific training, with exceptions only in the informal economy.
- Usually in Italy the trainings for the construction workers are organized within the vocational schools system, that for the vast majority in Italy are represented by the so called “Building schools” [28]. These institutes are recognized by the Italian law [39] and by the national joint associations and labor unions of the construction sector, representative of both the employers and the employees [28].
- We chose four different “Building schools”, two in the north of Italy and two in the south of the country, to retrieve a sample representative of the Italian panorama of the workforce in the construction sector. We considered the current distribution in the country of the workforce in the construction sector, about 79% composed by Italian workers and 21% of immigrant workers [29], and, as we were particularly interested in evaluating the ethnic background, we tried to recruit as much as foreign workers as possible, according to the exclusion and inclusion criteria of the study, finally resulting in the present research in a slightly higher presence of immigrant workers in the sample (27%) compared to the national data [29].

Another limitation of the study is related to the possibly insufficiently addressed issue of the language barriers, which are important to be considered when studying occupational aspects related to workers with different ethnic backgrounds. Unfortunately, as the questionnaire used was a written one, even if suitably prepared with a very simple language structure, we had to exclude the workers with an insufficient ability to read the Italian language. Actually, we collected with the general part of the questionnaire some information on the ability to understand and speak the Italian language, and it should be noted that the twelve items considered for the four dimensions of risk perception have been identified after an internal consistency and a factor analysis involving a larger group of items prepared by a multidisciplinary team of experts [24]. The first version of the questionnaire was the object of a pilot administration, in order to evaluate what were the items better understood by the immigrant workers and what were the items and the related dimensions more relevant for the assessment of their occupational risks’ perception [24].

A further limitation of the study is the possible underestimation of all the factors that may have affected the perception of the occupational risks in the sample investigated, as the different groups significantly differed for some characteristics. Considering age, this may explain the generally better risk perception of Italian workers; this factor was included in the multivariate logistic regression analysis as a confounding factor. Regarding educational levels, this is another factor with a significant difference between the groups and that can be implied in explaining differences in risk perception, we decided to not consider it as a confounder, as in our sample we found increased percentages of higher educational levels in immigrant workers compared to Italians. It should be noted that the organization of the school system is different in the various countries of origin of the subjects who participated to the study, and that the information was only subjectively collected. Moreover, it was also considered that a possibly higher educational level of the immigrant workers compared to Italians could be balanced by their lower ability in understanding Italian language and, consequently, by their reduced possibilities to obtain comprehensive information for the prevention of the occupational risks at the workplace. On the other hand, other factors that were significantly different between Italians and immigrant workers, as e.g., the type of work contract, and that may have played a role in explaining, also, the differences in risk perception that were appropriately considered as confounders in the multivariate model.

Finally, other factors that we did not appropriately consider may have an impact on the perception of the occupational risks, and can be significantly different among various ethnic groups: we think in particular to the habit of alcohol consumption, that can represent an issue for some immigrant populations while not for others [38,40]. 

## 5. Conclusions

In the present study, the risk perception of a group of Italian and immigrant construction workers attending vocational schools in Italy was evaluated with a questionnaire, according to four relevant dimensions, previously determined based on the scientific literature and on a pilot study. These four dimension, identified for their relevancy in appreciating possible differences in risk perception between workers with different ethnic backgrounds, are perceived behavioral control, danger perception, safety climate, and attitude towards safe actions.

The main results in the whole sample of construction workers highlighted the importance of working conditions such as haste, fatigue, and fear of losing the job, as negative predictors of a correct adoption of safe behaviors, identifying also a general underestimation of the possibilities of work-related accidents and of the importance of the use of protective equipment, and recognizing the supervisor in the construction site as an extremely important figure to promote an adequate safety climate in the company.

Considering the ethnic background, in our study three different groups of workers have been identified and compared with native Italians: North African, eastern European, and Balkan workers. It was found that the perceived behavioral control dimension was particularly relevant for workers from North Africa, who have reported a higher degree, compared to Italians, of fear of losing the job, of perceived haste at work, and perceived pressure imposed by the supervisors. Both eastern Europeans and workers from Balkans obtained quite high scores compared to Italians in the dimension attitude towards safe actions, identifying as “brave” the workers avoiding an adequate use of protective equipment at work and fatalistically accepting the possibility of work-related accidents. However, on the other hand, the former group of eastern Europeans showed also a lower perception, in general, of the dangers at work, whereas the second group reported, compared to Italians, a more positive opinion of the safety climate within their companies. As a conclusion, the results obtained suggest that ethnic background is a relevant aspect for the determination of occupational risk perception of the construction workers. An adequate consideration of these characteristics is strongly recommended when designing safety preventive initiatives, including safety training of the workers. The emergent information from the conducted analysis suggests the organization of tailored safety training, taking into account the peculiarities of each ethnic group, with the aim of improving correct risk perception of different working situations. 

## Figures and Tables

**Table 1:** The section “C” of the questionnaire for the evaluation of risk perception of construction workers considering the ethnic background according to four dimensions: Perceived behavioral control (PBC), Danger perception (DP), Safety climate (SC), Attitude towards safe actions (ATSA).

Item	Text	Scoring	Dimension
1	“I don’t comply with safety rules because my supervisor tells me that I have to work quickly”	0–1–2–3–4–5–6–7–8–9–10 (where “0” means: “I absolutely disagree with the statement” and “10” means “I absolutely agree with the statement”	PBC
2	“I don’t comply with safety rules because I am afraid to lose my job”		
3	“I don’t comply with safety rules because I am too much tired”		
4	“My job is dangerous”		DP
5	“My specific tasks at the construction site are more dangerous than other jobs in the construction sector”		
6	“I think it’s possible to be seriously injured at work”		
7	“My team leader always respects the rules to avoid risks at work”		SC
8	“If we respect the safety requirements, it’s possible to avoid occupational injuries”		
9	“My supervisor wants me to work with absolutely no risks of injuries”		
10	“One can expect to be seriously injured at work”		ATSA
11	“People working without protective equipment are brave”		
12	“I don’t comply with safety rules because I am brave”		

**Table 2:** Educational and general job characteristics (from section “A” of the questionnaire) in the whole sample and by ethnic groups. NB: values are reported as absolute numbers (and percentages—%—of the total sample).

		Region of Origin				
	Total (n = 572)	North Africa (n = 22)	Eastern Europe (n = 80)	Balkans (n = 53)	Italy (n = 407)	*p* Value
**Educational level**						<0.0001 *
None	4 (0.7)	0 (0.0)	1 (25.0)	0 (0.0)	3 (75.0)	
Primary school	274 (48.8)	11 (4.0)	15 (5.5)	15 (5.5)	233 (85.0)	
High school	259 (46.1)	10 (3.9)	60 (23.2)	33 (12.7)	156 (60.2)	
University	14 (2.5)	1 (7.1)	3 (21.4)	3 (21.4)	7 (50.0)	
Missing	11 (2.0)	0 (0.0)	1(9.1)	2 (18.2)	8 (72.7)	
**Professional Qualification**						*0.7697*
Construction manager	53 (9.4)	2(3.8)	2(3.8)	3(5.7)	46 (86.8)	
Foreman	28 (5.0)	0(0.0)	2(7.1)	1(3.6)	25(89.3)	
Generic worker	190 (33.8)	9(4.7)	35(18.4)	25(13.2)	121(63.7)	
Specialized worker	168 (29.9)	9(5.4)	21(12.5)	16(9.5)	122(72.6)	
No qualifications	26 (4.6)	1(3.9)	7(26.9)	1(3.9)	17(65.4)	
Other qualifications	78 (13.9)	1(1.3)	10(12.9)	5(6.4)	62(79.5)	
Missing	19 (3.4)	0(0.0)	3(15.8)	2(10.5)	14(73.7)	
**Type of work contract**						*0.0891*
Unemployed	51(9.1)	1(2.0)	11(21.6)	6(11.8)	33(64.7)	
Permanent employee	359(63.9)	10(2.8)	48(13.4)	30(8.4)	271(75.5)	
Seasonal worker	90(16.0)	6(6.7)	14(15.6)	7(7.8)	63(70.0)	
Craftsman (external company collaborator)	22(3.9)	1(4.6)	0(0.0)	1(4.6)	20(90.9)	
Other contract type	26(4.6)	3(11.5)	4(15.4)	5(19.2)	14(53.9)	
Missing	14(2.5)	1(7.1)	3(21.4)	4(28.6)	6(42.9)	

Differences among groups have been evaluated with Chi-square test (significant *p* value level: * <0.05).

**Table 3:** Information on work-related injuries and on their prevention (from section “B” of the questionnaire) in the whole sample and by ethnic groups. NB: values are reported as absolute numbers (and percentages—%—of the total sample).

		Region of Origin				
	Total (n = 572)	North Africa (n = 22)	Eastern Europe (n = 80)	Balkans (n = 53)	Italy (n = 407)	*p* Value
**Previous personal experience of occupational injuries**						<0.001 *
Yes	173 (30.78)	4 (0.71)	13 (2.31)	12 (2.14)	144 (25.62)	
No	374 (66.55)	16 (2.85)	67 (11.92)	41 (7.30)	250 (44.48)	
missing	15 (2.67)	2 (0.36)	0 (0.00)	0 (0.00)	13 (2.31)	
**Eyewitness of occupational injuries happened to a colleague**						
Yes	19 (3.38)	4 (0.71)	4 (0.71)	4 (0.71)	7 (1.22)	<0.001 *
No	495 (88.08)	15 (2.67)	74 (13.17)	49 (8.72)	357 (63.52)	
missing	48 (8.57)	3 (0.53)	2 (0.36)	0 (0.00)	43 (7.65)	
**Information received on the prevention of the risks of occupational injuries judged to be adequate**						
Yes	404 (71.89)	12 (2.14)	52 (9.25)	36 (6.41)	304 (54.09)	0.0170 *
No	107 1(9.04)	7 (1.25)	24 (4.27)	12 (2.14)	64 (11.39)	
missing	51 (9.07)	3 (0.53)	4 (0.71)	5 (0.89)	39 (6.94)	

Differences among groups have been evaluated with Chi-square test (significant *p* value level: * <0.05).

**Table 4:** Mean score (and SD) for each item of the section “C” of the questionnaire evaluating the four different dimensions of risk perception in the whole sample of construction workers.

Dimension of Risk Perception	Related Items of the Questionnaire	Mean Score ( ±SD) (0 = Absolutely Disagree- 10 = Absolutely Agree)
*Perceived behavioral control*	I don’t comply with safety rules because my supervisor tells me that I have to work quickly	6.9 ± 3.2
	I don’t comply with safety rules because I am afraid to lose my job	6.0 ± 3.6
	I don’t comply with safety rules because I am too much tired	5.8 ± 3.6
*Danger perception*	My job is dangerous	3.5 ± 1.6
	My specific tasks at the construction site are more dangerous than other jobs in the construction sector	7.5 ± 2.7
	I think it’s possible to be seriously injured at work	4.7 ± 3.2
*Safety climate*	My team leader always respects the rules to avoid risks at work	4.3 ± 3.5
	If we respect the safety requirements, it’s possible to avoid occupational injuries	8.9 ± 2.4
	My supervisor wants me to work with absolutely no risks of injuries	6.4 ± 3.5
*Attitude towards safe actions*	One can expect to be seriously injured at work	8.4 ± 2.6
	People working without protective equipment are brave	8.4 ± 2.3
	I don’t comply with safety rules because I am brave	4.4 ± 3.4

**Table 5:** Association between the scores obtained at each of the four dimensions of risk perception in the sample of construction workers and the different ethnicities. NB: the values reported in the table are Odds Ratios (and *p* values, considering a statistically significant level * <0.05), adopting Italians as a referent group.

Region of Origin	Perceived Behavioral Control	Perception of Dangers	Safety Climate	Attitudes Towards Safe Actions
North Africa	1.752 (0.040 *)	1.519 (0.070)	1.226 (0.140)	1.462 (0.070)
East Europe	1.033 (0.210)	0.635 (0.002 *)	0.914 (0.160)	1.441 (0.011 *)
Balkans	0.831 (0.096)	0.92 (0.174)	1.611 (0.005 *)	1.601 (0.007 *)

## Data Availability

Not applicable.

## Supplementary Materials

The following are available online at https://www.mdpi.com/2254-9625/11/1/8/s1, S1: The section “A” and “B” for the collection of general socio-demographic and occupational information of the questionnaire for the evaluation of risk perception of construction workers, considering the ethnic background.
