# Supplementary material for: Risk Perception and Ethnic Background in Construction Workers: Results of a Cross-Sectional Study in a Group of Trainees of a Vocational School in Italy
Source: Eur J Investig Health Psychol Educ. 2021 Jan 26;11(1):96–109. doi: 10.3390/ejihpe11010008 (PMC8314337; doi:10.3390/ejihpe11010008)
Supplement: Supplementary file 1 [file ejihpe-11-00008-s001.pdf]

General sections "A" and "B" of the questionnaire for the evaluation of the occupational risks perception of Italian and immigrant construction workers (NB: available in Italian language only)

QUESTIONARIO SULLA PERCEZIONE DEL RISCHIO

CODICE PROVINCIA:__________ **Codice SOGGETTO:**________________ Data: ________

Dimensione azienda
1) lavoratore autonomo
2) fino a 3 addetti
3) da 4 a 6 addetti
4) da 7 a 10 addetti
5) da 11 a 30 addetti
6) più di 30 addetti
7) non ho contratto
8) ancora non lavoro

(pag. 1-2 in 4 lingue: *italiano, inglese, francese, arabo*)

Descrivi QUALI COSE FAI DURANTE LA TUA GIORNATA DI LAVORO
(es. costruzione muratura, tagliole, assistenza, carico e scarico tubi e giunti dei ponteggi, ecc., oppure anche altri lavori come es. verniciatore, meccanic, autista)

____
____
____

Sezione A, Inquadramento

1) Qual è il tuo paese di origine?

2) Da quanti anni sei in Italia?
1) Meno di 1
2) 1-3
3) 4-5
4) 6-10
5) Più di 10
6) Solo per italiani: da sempre

3) Qual è la tua qualifica professionale del lavoro che stai facendo adesso o che hai fatto in passato?
1) Capocantiere
2) Caposquadra
3) Operaio generico
4) Operaio specializzato
5) Attualmente sono senza lavoro
6) Altro(specificare)
....

4) Quanti anni hai? __________________ (anni già compiuti)

5) Il tuo rapporto di lavoro è:
1) Adesso sono senza contratto
2) Ho un posto fisso
3) Lavoro per poco tempo (es. 3/6 mesi)
4) Come artigiano
5) Altro ....

**6) Che scuole hai fatto?**	1) Nessuna<br>2) Primarie<br>3) Superiori<br>4) Università
**7) Da quanti anni lavori nell'edilizia?**	1) Non ho mai lavorato in edilizia<br>2) Meno di 1<br>3) 1-3<br>4) 4-5<br>5) 6-10<br>6) Più di 10

**8) Che lavoro fai di più?**<br>**<u>Scrivere una sola risposta</u>**	1) Muratore<br>2) Carpentiere<br>3) Pavimentatore<br>4) Idraulico-elettricista<br>5) Lattoniere<br>6) Intonacatore<br>7) Montatore ponteggi<br>8) Ferraiolo<br>9) Gruista<br>10) Movimento terra<br>11) Rimozione amianto<br>12) Jolly<br>13) Stradino-asfaltatore<br>14) Altro<br>(specificare)____________________
**9) Hai frequentato almeno un corso di formazione in aula sulla sicurezza ?**	SI NO

10)Se si, quante ore di corso in aula hai fatto?

1) Meno di 8
2) Tra 8 e 16
3) Tra 16 e 24
4) Più di 24

<u>Solo per gli stranieri</u>

**11) Capisci l'italiano?**	SI	NO
**12) Riesci a leggere in italiano?**	SI	NO
**13) Riesci a parlare in italiano?**	SI	NO
**14)Riesci a scrivere in italiano?**	SI	NO
**15) Quando lavori parli in italiano con i tuoi compagni di lavoro?**	SI	NO
**16) Quando lavori parli nella tua lingua d'origine con i tuoi compagni di lavoro?**	SI	NO

<u>NB) Se riesci a leggere e a scrivere in italiano continua la compilazione del questionario altrimenti fermati</u>

Sezione B, Dati generali

**17) Ho avuto almeno un incidente sul lavoro in Italia**	SI NO
**18) Ho avuto almeno un incidente sul lavoro all'estero**	SI NO
**19) Almeno una volta ho visto un mio compagno farsi molto male sul lavoro** .	SI NO
**20) <u>Se hai risposto SI alla domanda di prima (19)</u>, Dopo averlo visto ho cambiato il mio modo di lavorare per paura di farmi male**	SI NO
**21) Il mezzo di trasporto che utilizzo di più per raggiungere il posto di lavoro è:** ***Scrivere una sola risposta*** . 1) bicicletta 2) moto 3) automobile 4) mezzo pubblico (es. autobus/treno) 5) mezzo aziendale (es. furgone)	
**22) A volte quando rientro dal lavoro alla sera sono stanco e faccio più fatica a guidare.** .	SI NO
**23) Ho avuto almeno un incidente mentre andavo o tornavo da lavorare** .	. SI NO
**24) Quando lavoro su un tetto, mi sento più sicuro se:** 1) c'è un ponteggio di protezione contro le cadute 2) uso solo i dispositivi di protezione come cinture di sicurezza o altro	
**25) Ho avuto abbastanza informazioni sui rischi di infortunio del mio lavoro** .	. SI NO
**26) Ho avuto abbastanza informazioni su cosa devo fare per non farmi male durante il lavoro**	SI NO
**27) Le informazioni sulla sicurezza me le dà a voce il mio padrone** .	. SI NO
**28) Le informazioni sulla sicurezza me le dà a voce il mio capocantiere/caposquadra**	SI NO
**29) Le informazioni sulla sicurezza me le danno con dei fogli scritti** .	. SI NO
**30) Le informazioni sulla sicurezza me le danno con una lezione in aula** .	. SI NO
**31) Il modo in cui preferisco avere informazioni sulla sicurezza e salute del lavoro è:** **(Scrivere solo una risposta)** . 1) a voce dal capo 2) dal medico 3) con un testo scritto 4) con una lezione 5) con dimostrazioni pratiche	
